# The History, Diagnosis, and Treatment of Typhoid and Typhus Fever; with an Essay on the Diagnosis of Bilious Remittent and Yellow Fever

**Published:** 1844-04

**Authors:** 


### Art. V.

*The History, Diagnosis, and Treatment of Typhoid and Typhus Fever; with an Essay on the Diagnosis of Bilious Remittent and Yellow Fever.* By Elisha Bartlett, m.d., Professor of Medicine in Transylvania University.—*Philadelphia*, 1842. 8vo, pp. 394.

This work has been written, as the author informs us in his preface, to supply what he regards as an acknowledged want in the medical literature of the United States,—a treatise on fever which shall be a safe guide to its practitioners. The British works on the subject, such as those of Drs. Armstrong, Southwood Smith, and Tweedie, are not adapted to this purpose, from the material circumstance that the fever which chiefly prevails in this country, and whence, consequently, British writers have drawn their descriptions, is, according to Dr. Bartlett, specifically different from that found in the United States; and, according to other observers, varies, at least, considerably from it; whilst the work of Louis, of which America possesses a translation from the pen of Dr. Bowditch, though constituting one of "the few imperishable monuments reared in the pathway of our science," is not adapted to the wants and tastes of the majority of practical men.

The form of continued fever prevailing in the United States, the author shows to be that to which Petit and Serres, in 1813, gave the name of Entero-mesenteric Fever; which Bretonneau has called Dothinenterite; Cruveilhier and others, Follicular Enteritis; the Germans, very appropriately according to our opinion, Abdominal Typhus; and Louis, Gerhard, Jackson and others, Typhoid Fever. The history of this fever—its symptoms, anatomical character, diagnosis, mortality, theory, treatment, and definition, occupy the first part of the volume. This portion is very creditable to the observation, judgment, and literary research of the writer. The Second Part comprises the history of typhus. This is avowedly less original than the first part; its materials being derived chiefly, as its writer admits, from British physicians, especially those of Ireland and Scotland.

These varieties, or, as Dr. Bartlett would say, species of fever, were so fully discussed in former numbers of this Journal,* that we do not feel ourselves called upon to examine at any considerable length these two portions of the work. We might, indeed, have passed them over with that general note of approbation which they unquestionably merit, had there not been mingled in them some controversial matter addressed to ourselves. Into this we do not purpose entering at any considerable length; but to pass it entirely unnoticed might be interpreted to imply either want of confidence in our own opinion, or of respect for the authority of Dr. Bartlett; and we are possessed with neither of these feelings. It will be manifest, from the annexed extract, that we have no occasion to complain of the spirit of the author's criticism. The candid advocacy of truth, or what we conceive to be truth, is our quarterly vocation; and far be it from us to object to others labouring in our own very creditable calling.

"I shall conclude this historical survey of facts and opinions, bearing on the

* See British and Foreign Medical Review, vol. XII, pp. 22 and 294.

question of diagnosis before us by a short reference to an article contained in the July and October numbers of the 'British and Foreign Medical Review' for 1841. This article contains a pretty full exposition of the subject under consideration, and abating some mere smartness in its criticisms of Christison, Gerhard, Lombard, and Starbach, it is written in a good spirit, as well as with fairness and ability. Its noble tribute to Louis has already been noticed. The writer of the paper, after an examination of all the accessible and valid evidence in the case, comes to the conclusion, that the contagious typhus of Great Britain and the typhoid fever of France are different varieties, only, and not distinct species of disease. I have already gone over nearly all the ground occupied by this writer; I shall have occasion, therefore, to notice only two or three of his statements and opinions. The most important of these, in its connexion with the diagnosis of the two diseases, is this: in his tabular comparison of typhoid fever and typhus, he sets down, so far as the abdominal lesion is concerned, as typhus, all the cases of fever occurring in Britain; thus settling, beforehand, the very question at issue, in relation at least to one of its elements. The writer admits, that the two forms of fever may generally be distinguished during life; but alleges that there are cases in which such distinction cannot be established. The number and authenticity of these latter is certainly, thus far, very limited; and if a difference of symptomatology, sufficiently marked to be generally and readily recognized, corresponding constantly with a most important difference in the state of certain organs found in fatal cases, is not adequate to constitute separate diseases, it is not easy to see in what radical nosological distinctions are to be found. In order to account for the great differences in the appearances of the eruptions of the two diseases, the reviewer suggests the hypothesis, certainly improbable and gratuitous enough, that the lesions of the skin and of the intestine may be supplementary of each other; a most facile method, assuredly, of disposing of a difficulty." (pp. 308-9.)

Dr. Bartlett is not correct in stating that we have, in the tabular view he refers to, set down, so far as the abdominal lesion is concerned, as typhus, all the cases of fever occurring in Britain. The question we endeavoured to decide was, that of the identity or non-identity of the fever of France and Great Britain; and for the attainment of this object we placed in parallel columns the symptoms, anatomical lesions, &c., observed by the writers of the two countries on their respective fevers, and the heading of the table showed that such was our intention in its construction. We were holding up to view facts as presented to us by the best writers in both countries. We were not regarding anything as decided, but were furnishing materials for decision. Our classification throughout was into British and French, not typhus and typhoid. That we were not irrespective of facts tending to identify a proportion of the cases occurring in England with "the typhoid form," is manifest from the following remarks in the table, under the head of anatomical characters: "A special lesion of the patches of Peyer is of extremely rare occurrence, as a general proposition; but in almost all, or in actually all the cases examined at certain periods of the year, or in certain situations that lesion is discovered. When it does exist, the phases through which it passes, and the influence it exercises on the mesenteric glands, are the same as in France." (Brit. and For. Med. Rev., vol. XII, p. 324.) We certainly stated that the form of continued fever, attended with disease of the ileum, may be commonly distinguished by its *local abdominal symptoms* from a fever not so attended; and the author, omitting the words we marked in italics, and connecting this unqualified statement with the general detection of the Peyerian lesion in those examined after death, exclaims, "if a difference of symptomatology sufficiently marked to be generally and readily recognized, corresponding constantly with a most important difference in the state of certain organs, found in fatal cases, is not adequate to constitute separate diseases, it is not easy to see in what radical nosological distinctions are to be found." To this we would in the first place reply,—that any one can distinguish, at a glance, confluent from distinct smallpox; but no one, therefore, regards these diseases as specifically different. With regard to the argument derived from pathological anatomy, it appears to us to be divested of its weight, in great measure, by Dr. Bartlett's own statements:

"I do not think," he says, "that we are justified in referring typhoid fever, considered as a disease, as an integral, though complex pathological condition and process, or series of processes, to this single local lesion of the intestines. It seems to me much more satisfactory and philosophical, much more in accordance with what is seen in many other diseases, to look upon the lesion of the elliptical plates, not as the local cause of all the other appreciable phenomena of typhus fever, but as constituting one of the pathological elements in a very obscure and complex disease; all which elements, and this quite as much as the others, are themselves the result of some morbific agent, or influence, or process, the nature, sources, and operation of which are wholly unknown to us." (pp. 74-5.)

In a subsequent passage in relation to the view which regards typhoid fever not as an essential or idiopathic fever, but an enteritis, or a follicular enteritis, or a dothinenteritis, and assigns to it a nosological position among the local phlegmasiæ, he remarks:

"The most obvious objection to this view of the nature of typhoid fever consists in the circumstance, that there is no uniform proportion between the extent of the local disease and the severity of the symptoms. There are many fatal cases in which the intestinal lesion is very limited in extent; there are others where the whole character of the disease has been unusually mild, and in which, when life has been destroyed by some secondary and accidental complication, the alteration of the intestine has been found to be very extensive and profound." (Bartlett, p. 141.)

We are not yet convinced that this one of the elements of a very complex disease, and an element between the extent of which and the general severity of the disease there is no proportion, granting that it is almost invariably existing, (Dr. Bartlett labours to prove that it is, without qualification, invariable, but, we think, with more skill than success,) should be considered to establish an impassable barrier, a broad and specific distinction between the fevers existing in France, the United States, and other parts of the world, and one prevailing in this country, bearing in all other respects—etiology, mode of invasion, symptomatology, and general course—the closest possible relation to it, and frequently commingled with it during the same epidemic in the wards of the same hospital.

We remain, then, of the opinion that we expressed some time ago,* that the continued fever of England and France, and we would add too, of America, are the same species of disease, but that they are different varieties of this species; and we find our opinion confirmed by the Report of M. Landouzy on the epidemic in the jail at Rheims, a report quoted at great length by Dr. Bartlett, and by which he admits that some doubt is thrown on the specific difference of the two diseases.

The epidemic in question was confined to the inmates of a certain quarter of the prison at Rheims, and prevailed between the 1st of Octo-

* See British and Foreign Medical Review, vol. XII, p. 326.

ber, 1839, and April, 1840. The entire number of cases was 138, 103 of which were amongst the inmates of the prison; the remaining 35 consisting of physicians, medical students, nurses, and others connected with the hospital where the patients were treated. An important point connected with these cases is, that they all came under the observation of the medical attendants immediately on the commencement of the disease. Among the first symptoms was stupor, which frequently showed itself as early as the second or third day, and continued until it was lost in coma or delirium. It differed from mere somnolence and coma. The expression of the countenance was that of half-demented and stupid astonishment. It was the *stupor attonitus* of Foes. In half the cases it was strongly marked, in the other half it was slight in degree. True somnolence and coma appeared in a certain number of cases, later in the disease, often about the tenth day. Profound coma, so that the patient could not be roused, existed in only twelve cases. Delirium was very common, usually making its appearance between the third and the eighth day. It was generally low and muttering in its character, and, in fatal cases, it continued until death. Headach was uniformly present at the commencement of the disease. It was, for the most part, dull and heavy, and felt especially over the eyes. It continued for an uncertain period of time, gradually disappearing or losing itself in coma or delirium. *Subsultus tendinum* was common, and strongly marked in grave cases. Redness of the eyes, *tinnitus aurium*, and deafness were present in a certain proportion of cases, but differed in no obvious particulars from the same symptoms in typhoid fevers. There was great loss of muscular strength, from the beginning of the disease. In every case except the first, which was not carefully examined, there was an abundant cutaneous eruption, consisting of small spots, or *ecchymoses*, of a red, violet, or black colour, not elevated above the skin, and not disappearing on pressure. They were always found on the chest, often also on the abdomen, and in some cases they extended to the arms and legs. They commonly showed themselves about the fourth or fifth day, and gradually faded away between the tenth and the eighteenth. They were abundant and confluent in proportion to the gravity of the disease. The bodies of the sick exhaled a strong, offensive odour, resembling that of mice. In regard to the absence of appetite, to thirst, the state of the lips, tongue, and mouth, nothing special was observed, differing from what occurs in typhoid fever. Nausea was present at the commencement of the disease in all the cases. *Meteorism and abdominal pains were uniformly absent.* There was diarrhea in the beginning of the disease in only four cases. In all the others there was no apparent disturbance in the functions of the intestinal canal. The bowels were more inclined to constipation than to looseness. A distinct, well-marked, sibilant rhonchus was present in all the cases. Epistaxis occurred in eight cases. The temperature of the surface was uniformly elevated; the heat was dry and burning. In no instance was there gangrene of any part of the body. In the six autopsies which were performed, the intestinal lesions characteristic of typhoid fever were present. The elliptical plates were either thickened and elevated or they were the seats of ulcerations; and the mesenteric glands corresponding to them were enlarged. *The spleen was not increased in size in any of the cases:* in four it seemed somewhat softened.

Here we have the symptoms which every practitioner in Great Britain would describe under the name of typhus gravior, jail, or petechial fever; and originating under circumstances which give rise to such fevers in England; for we are informed that the quarter of the prison was originally intended to accommodate from 80 to 100 inmates, but at the time when the epidemic appeared its population amounted to 180. Yet we are told to regard it as something specifically distinct from typhus, because the plates of Peyer are, on examination, found diseased. The points of resemblance are multitudinous, but they avail nothing, because the one (supposed) point of discrepancy sitteth in the iliac region. Dr. Bartlett suggests, by way of surmounting the difficulty, that the causes of typhus and typhoid fever were commingled, and gave rise to a hybrid progeny, formed of the elements of two dissimilar diseases. M. Landouzy, with much prudence, says that we must await the result of ulterior observation before we shall be able to settle definitively the question regarding the identity of these several forms of fever. "In effect," he adds, "if in all future epidemics of the typhus of camps, of jails, of hospitals, &c. we find, as in that of Rheims, complete absence of disease of the spleen, and great differences between the symptoms and those of typhoid fever, we must confine ourselves to the conclusion that typhus and typhoid fever are analogous and not identical diseases. If, on the contrary, we find that in one epidemic diarrhea is absent, in another the petechial eruption, in another the rose-spots, and so on, we must conclude that these differences depend only on variations in the action of the epidemic cause, and that the disease is, in its nature and essence, identical with typhoid fever."

For the final adjustment of this question much will justly be expected from British writers; for we consider that a great deal of the confusion in which it is still involved has arisen from the vague and, we would add, too, declamatory character of some of the most popular British works on fever—works apparently more calculated to display the eloquence of their authors than the precise character of the fever of this country. The repair of the evil which we have done will be looked for at our hands. The world does not possess such a history of British fevers as Louis has furnished to those of France, or even as Dr. Bartlett has, in the present volume, given of those of the United States; and the decision of the question of identity and discrepancy has been attempted from unequal evidence—that from other parts of the world being complete and precise, that from Great Britain imperfect and vague. We beg, in concluding this branch of our subject, to express the belief that in proportion as it is duly investigated, the more will British be found to resemble continental fever; and, as furnishing some ground for this belief, we would remark, that all the cases which we have had examined during the current year (1843,) have displayed the triple lesion, affection of the plates of Peyer, that of the mesenteric glands, and enlargement of the spleen. These were all of them cases of young persons, or of persons, at least, under forty years of age. Among symptoms during the same period, we have observed diarrhea, meteorism, and pink-coloured, lenticular spots, not passing into the petechial state, to be attendants on a majority of the cases. Abdominal pains have been frequent, but not invariable; hemorrhage from the intestines has existed in one half of the cases, and in the fatal cases has been profuse. We would remark, that in some parts of England hemorrhage from the intestines has, of late years, been a very frequent accompaniment of fever, and has often appeared to be, by its profuseness, a cause of death.

Dr. Bartlett is of opinion that the common continued fever of Armstrong and the slow nervous fever of Huxham are identical with typhoid fever. We cannot agree with him in this opinion, if by typhoid fever he means this disease in its ordinary intensity; for this were to identify a very fatal disease with one that is rarely so. We have long had reason to think that the disease described by Armstrong, and very generally by medical men in this country under the name of common continued fever, which we believe to be the same as the low nervous fever of Huxham, is a mild form of either variety of typhus. We have seen a member of a numerous family sicken of this common continued fever, and pass easily through it, whilst successive members of the same family have been attacked with typhus in its most aggravated form. Under similar circumstances we have seen what, from symptoms, would be called the typhoid variety arise in families, and spread from an original in appearance equally slight. It should ever be borne in mind, that all epidemics have a wide range of severity. Smallpox, scarlatina, measles, cholera may be an intense and dangerous disease, or a slight ailment; and typhus forms no exception to this law: but no little confusion has prevailed, and the same disease has passed under various names, because it has been forgotten.

Admitting the accuracy of the principle of Rousseau, that truth is in the things surveyed, and not in the mind which surveys them, and that all has not yet been done which is required to elicit the whole truth from the fevers of this country, we yet will venture to prognosticate that the ultimate issue of the investigation into their nature which is evidently now in progress, will show the truth of a remark of Dr. Southwood Smith, that "there is but one kind of idiopathic continued fever;" and that confusion has arisen from regarding varieties in degree and complications as distinct diseases.

The Third Part of the volume is dedicated to the diagnosis of bilious remittent fever from "the two continued fevers," on the one hand, and from yellow fever on the other. We think the first branch of this subject not devoid of interest in this country; for in certain parts of it remittent fever frequently prevails; whilst, in districts which it does not habitually visit, it appears occasionally in seasons of unusual warmth, and want of familiarity with its character causes it to be misunderstood by resident practitioners. Confusion and controversies have, within our remembrance, arisen from the confounding of bilious remittent with typhus.

The symptoms by which we are taught to discriminate these two diseases are indicated by Dr. Bartlett, as our own experience enables us to state, with great skill. As might be supposed, there are symptoms common to the two diseases, yet even in this case, there is a difference in the degree and form of these symptoms as they appear in the respective affections. Thus both have chills or rigors, but those attending remittent are more formal, and show a greater tendency to recurrence at intervals of twenty-four or forty-eight hours, the quotidian or tertian type, than is observed in those accompanying typhus. The temperature in remittent varies more considerably than in typhus, rising and falling with the exacerbations and remissions of the disease, and, during these last, being either natural or below the healthy standard. The pulse in remittent is less rapid than in typhus. In eleven cases, in which its frequency was noticed by Dr. Gerhard, it exceeded a hundred in the minute in only two. Its condition, too, is more variable in the one disease than the other, being considerably accelerated during the exacerbations of remittent, and falling often to its natural standard in the interval. "One of the most striking and invariable differences between remittent and continued fever consists in the state of the mind. In the former disease it is rarely affected to any appreciable extent. Of eleven cases, observed by Dr. Gerhard, in 1834, at the Pennsylvania Hospital, all terminating in recovery, there was slight delirium in one only." The tongue in remittent is generally moist, and covered with a thick fur, of a whitish or yellowish or yellowish-white colour; and it begins to clean earlier than in continued fever. Nausea, vomiting, or both, are very constantly present in remittent fever; whilst diarrhea, so common in one form of typhus, is very rare. True tympanitic distension of the abdomen is seldom found in remittent; but pain, tenderness on pressure, with a feeling of weight and oppression, and fulness extending across the epigastrium from one hypochondriac region to the other are of frequent occurrence. The pain and soreness are more frequent in the left than in the right hypochondrium, a circumstance ascribed to the enlarged and congested state of the spleen.

There are symptoms common in typhus, which are never seen in remittent, as the rose-coloured spots so frequent in one form, and the dusky petechiæ so general in the other. In a few cases of remittent, there are sudamina mostly confined to the neighbourhood of the neck, but never any other cutaneous eruption.

The pathological appearances in this disease have been in general more vaguely described than those in typhus; but sixteen cases were examined by Drs. Gerhard and Stewardson in the Pennsylvania hospital with great precision; and the appearances have been accurately reported. The stomach in five of six of these cases displayed traces of previous inflammation. These consisted of mamelonation, and changes in the thickness, density and colour of the membrane variously combined in different cases. The mucous membrane both of the small and large intestines, was generally free from any considerable alteration; only such accidental lesions being found as are common after death, in most acute diseases. The elliptical plates were found uniformly healthy. In all the six cases, examined by Dr. Stewardson, in which the duodenum was particularly noticed, the mucous follicles or glands of Brunner, were very distinct and prominent. The liver was found of the colour of bronze, or a mixture of bronze and olive; in one case, it was found of a dull lead colour externally, internally bronzed with a reddish shade; in another between a brown and an olive, the latter predominating; and, finally, of a pale slightly greenish lead colour, with a tinge of brown in one instance. This peculiar alteration in the liver, Dr. Stewardson is disposed to consider the essential anatomical characteristic of remittent fever, although he admits that his cases have not been sufficiently numerous to determine the point definitively.

The spleen was constantly altered in remittents. It was softened and enlarged, and generally to a great degree. It was often found of dark blue or black colour, and of a pulpy consistence, the aspect of the organ being frequently very much that of a sac containing black, clotted, venous blood. This is precisely the condition of the spleen which we have ourselves constantly found in malignant intermittents, the *febres algidæ* of Torti and others. In remittent the mesenteric glands are free from disease.

After a recapitulation of the diagnostic marks between remittent and typhus or typhoid fever, Dr. Bartlett remarks :

"In some rare instances, where the disease is protracted, where the remissions are obscurely marked, where the tongue becomes dry and brown, where there is some delirium or stupor, and where other indications of the typhoid state show themselves, there may be some difficulty in forming a positive diagnosis. But even under these circumstances, the previous history of the case, and absence of many of the symptoms of typhoid and of typhus fever will prevent us from mistaking the disease for either of these latter affections." (p. 350.)

How well does our own matchless Sydenham indicate this difficulty and the scrutiny by which it is to be surmounted :

"Licet nonnunquam earum aliquo de Intermittentium naturâ reverâ participent, nullo charactere admodum visibili easdem prodente. Ut cum præmaturè, Julio mense v. gr., Intermittentes autumnales ingrediuntur atque increbescunt, non statim genuinum typum inducunt (quod Intermittentibus vernis quidem solemne est) sed *continues Febres* ita per omnia imitantur, ut nisi *castigatissimo utrasque examine trutinareris*, ab invicem *discriminari* non possint; at retuso paulitim constitutionis impetu, et frænatâ vi, jam in typum regularem migrant; atque exeunte autumno, larvâ abjectâ, Intermittentes se esse, quales ab initio reapse fuerunt, palam fatentur, sive Quartanæ illæ fuerint, sive Tertianæ; quod si non diligenter animadverterimus, cum magno ægrorum nostrorum malo medicantes hallucinabimur, dum hujusmodi febres, quæ ex Intermittentium numero sunt, pro continuis veris et genuinis habeantur."*

Dr. Bartlett argues, we should say needlessly, in favour of the essential identity of remittent with intermittent and congestive fever, this last being the somewhat hypothetical name given in the United States to the *fièvres intermittentes pernicieuses* of Bailly and other French writers, and the *febres intermittentes malignæ* and *algidæ* of the Italian writers, those fevers of which the Pontine marshes furnish so abundant a supply to the hospital of Santo Spirito at Rome. His reasons are, the general correspondence of symptoms in the three forms of disease, and especially in the community to all of them of the remarkable phenomenon of periodicity, and, moreover, the accordance among them all as to pathological conditions. He quotes the following account of these conditions observed by Dr. Gerhard in the congestive fever of America, and points out its agreement with that given by Bailly in his elaborate essay on the malignant intermittents of Rome :

"In all these cases, the glands of Peyer, as well as the other intestinal follicles, were found perfectly healthy. The large intestine was occasionally, but not constantly diseased, whilst the stomach, and, to a still greater degree, the liver and spleen, were invariably found in a morbid condition. If the fever proved fatal in the course of the first fortnight, the liver and spleen were softened as well as enlarged; but if the disease assumed a more chronic form, the viscera were hardened as well as hypertrophied. The results of late examinations have confirmed those already obtained, and showed that the follicles of the small intestines are free

* Opera Universa, ed. 1726, p. 44.

from lesions, and that the anatomical character of the disease is to be looked for in the spleen, liver and stomach." (Bartlett, p. 352.)

The strongest argument, however, for their identity is that they are severally convertible, one into the other. A case of ordinary remittent fever is frequently changed in the natural progress of the disease, or by the effects of remedies, into a simple intermittent; or it may pass, as Dr. Bartlett remarks, in the other direction, by the supervention of the congestive elements in pathology, into a case of malignant or intermittent or remittent. How often have we ourselves seen the disease, which on the banks of the Gambia, or some other point in Africa, had existed as malignant or congestive remittent, yet had spared the life of the patient, converted in the course of his voyage to Europe into a simple intermittent promptly curable by preparations of cinchona. In the great influence exerted by such preparations over all the three forms of disease, Dr. Bartlett finds a strong corroboration of his arguments for their essential identity.

The essay on yellow fever opens with a disclaimer on obvious grounds of the necessity of establishing any diagnosis between this disease and typhus or typhoid fever. The discrepancies between the diseases are too manifest to require to be indicated. That the distance between this disease and remittent fever must require more attention is manifest, since in the days of Rush and Bancroft the opinion that yellow fever was merely a high and malignant degree of the common bilious remittent, was entertained not only by these writers, but was the general creed. One writer, however, of the period, Dr. W. Currie, who published a little work on the subject in Philadelphia, in 1798, expresses his conviction that they are distinct diseases in the following forcible and figurative language: "If we go down," says he, "to the meadows and marshes on the flats of the Delaware and Schuyskill, and look for yellow fever among the diseases which exhalations engender, it is not there. Imaginations and her whimsical daughter, theory, have circulated something in those places, which they have called its likeness; but the sallow imp of the marshes is the offspring of different parents, and differs essentially in its character from the jaundiced-eyed fiend, which extends its destructive sway by contagion."*

The decisive tone, however, of this passage did not set the question at rest, for when yellow fever appeared thirty years after (in 1828,) at Gibraltar, the question was, as it had previously been in the same place in other varieties, has "the jaundiced-eyed fiend" been imported from the West Indies, or is this merely an aggravation of our ordinary bilious remittent? The next coming winter extinguished the epidemic, but not the war of letters and pamphlets which it had engendered.

The following are the diagnostic marks selected by Dr. Bartlett:

"It does not appear that there is in yellow fever anything like the quotidian or tertian paroxysm and remission, which so generally characterize the several forms of remittent fever. Dr. Rufz, in speaking of the yellow fever of Martinique, in 1838, says, that there were no intermissions, and that the exacerbations were hardly perceptible. The yellow discoloration of the skin, in fatal cases of yellow fever, is more constant and more strongly marked than in those of remittent fever.

* Observations on the Causes and Cure of Remitting or Bilious Fevers, &c., Philadelphia, 1798, p. 43, and Bartlett, p. 357.

The headach would seem to be somewhat more intense, while acute local pains in various parts of the body are more common, and the general restlessness is more distressing in the former than in the latter disease. There is not often any considerable degree of delirium in either of the diseases, but stupor and coma are more frequent in the high grades, and in the congestive form of remittents, than they are in yellow fever. The early and almost invariable red suffusion of the eyes, constitutes a peculiar and very characteristic symptom of the latter disease. The great preservation of muscular strength, even up to the close of life, which is often so striking a phenomenon in yellow fever, does not appear to be common in remittent. Although the symptoms connected with the digestive apparatus are very constant and prominent in both diseases, they are far from presenting the same characters in each. To say nothing of the state of the tongue, which does not appear to be precisely the same in both fevers, there are very marked differences in relation to the vomiting. In remittent fever this is generally one of the earliest symptoms showing itself often on the first day of the disease. This is the case, also, not unfrequently in yellow fever, but in a larger proportion of instances, vomiting is not present till a later period, and in many fatal cases it does not appear until the last day or two of life. Besides this, there is the striking peculiarity of the matter vomited, at least in a large proportion of the fatal cases of yellow fever, consisting of the dark or black liquid which has already been described, and which is rarely witnessed in the course of remittent fever. Although the stools in the latter disease are frequently variously coloured, especially after the use of mercurial cathartics, I am not aware that they have often that peculiar black shade, which they present in many cases of yellow fever which are attended by the black vomit. Finally, pain and tenderness of the epigastrium are very uniformly present in both diseases, but the tension and fulness from one hypochondrium to the other, with the pain and soreness over the region of the spleen, which have been especially noticed by Dr. Stewardson, in remittent fever, seem to be less strongly marked, and have at least excited less attention in yellow fever." (pp. 371-3.)

Dr. Bartlett admits, and we agree with him, that there is not here any pathognomonic symptoms. The differences between the two diseases are in the degree only in which certain symptoms exist, excepting in the case of such as are not of *invariable* occurrence in yellow fever, as the red suffusion of the eyes, and black vomit. We are convinced, however, that if yellow fever were to make an irruption on any point where bilious remittent is the endemic disease, medical men would, after the occurrence of a case or two, perceive that they had something unwonted to deal with, and, even should they never have seen it before, would speedily discover the real title of their unwelcome guest. This is the more likely to be the case, since the first cases on the invasion of a new locality by yellow fever, or other epidemic diseases, are generally intense, and distinctly marked. The slighter cases which occur when the epidemic is on the decline, resemble more in character the ordinary endemic of the country; but, in such cases, preceding occurrences are a security against confusion. The respective duration of the diseases is a ground of distinction so broad and palpable, that it can scarcely fail to arrest the attention of the observer. The mean duration of remittent fever is shown by Dr. Bartlett to be a fortnight, whilst yellow fever, whether terminating favorably or the contrary, runs its course with great rapidity, the largest number of deaths taking place from the fifth to the seventh day inclusive; and the question of life or death in the disease being generally settled within the first week.

In his account of the pathological appearances, the author relies mainly on the work of Louis, the publication of which was directly owing to his able countryman, Dr. Shattuck, junior, of Boston. This work, and the pathological facts it contains, having already received ample notice in our pages,* we deem it unnecessary to go over the same ground again. The characteristics of the disease set forth by Louis, however, having been deduced from one epidemic, that at Gibraltar, in 1828, it is interesting to remark how far they are confirmed or otherwise, by transatlantic observation.

It will be in the remembrance of our readers that in the cases observed by Louis at Gibraltar, the liver was found to be invariably the seat of a remarkable lesion, consisting in a change from its natural colour. The colour assumed is described by Louis as being sometimes that of fresh butter; sometimes that of straw; sometimes that of coffee and milk; sometimes is a yellow gum colour; or a mustard colour, or the colour of sole leather; or, finally, as an orange colour. This change of colour extended, in most cases, throughout the whole substance, although more marked and uniform in the left than the right lobe. With this discoloration of the organ there was more or less of dryness; it did not contain its usual quantity of blood.

It is interesting to notice the confirmation of these views of Louis, gathered by Dr. Bartlett from earlier or distant observers. Dr. Chisholm, in his account of the yellow fever which prevailed in the West Indies, in 1793, speaks of the liver as being "of a colour nearly approaching to buff, or a mixture of yellow and that of ashes." This same appearance has since been noticed by Dr. Rufz, of Martinique. Dr. Palloni, in an account of the yellow fever at Leghorn, in 1804, says, "the external surface of the stomach, liver, and intestines, had a livid yellow colour." In a case examined, in 1841, by Dr. Stewardson and our author, the colour of the liver, throughout its whole extent, was like that of powdered gamboge or yellow ochre. Dr. Ashbel Smith, a writer we formerly noticed,† found the organ, in three cases out of seven, of a very light drab colour externally and internally, and destitute of blood.

The following is the author's comparison of the anatomical lesions found in yellow and remittent fever respectively:

"The alterations in the brain are alike slight and unessential in both diseases. The state of the blood has not been ascertained with sufficient accuracy to render it of any service in diagnosis. There is no important difference in the changes of the mucous membrane of the stomach in the two fevers; and in both, at least in a very large majority of cases, there are indubitable evidences of preceding inflammation. The differences in the state of the mucous membrane of the intestines are not constant and considerable enough to require any notice here. The substance of the lungs, in a moderate proportion of cases of yellow fever, presents a peculiar lesion, which, so far as I know, has never been observed in remittent fever; and which is indeed an exceedingly rare occurrence in any other disease." [This lesion is described by Louis to consist either of black spots, of from two to five lines in diameter, or masses of the same colour more or less impermeable to air. Usually they could be easily broken down; in some cases they yielded, by pressure, the blood of which they were almost entirely composed.‡] "The contents of the stomach and intestines, in yellow fever, differ very constantly and strikingly

* See British and For. Med. Review, vol. X, pp. 494-508. † Ib. vol. XII, p. 433.

‡ Louis on Yellow Fever, translated by Shattuck, p. 64, and British and Foreign Med. Review, vol. X, p. 497.

from those of the same organs in remittent fever. The most invariable and important differences, however, are to be found in the condition of the liver and spleen. The liver in remittent fever is commonly softened, and of a bronze or olive-gray colour; in yellow fever it is destitute of its usual quantity of blood, and of a bright yellow colour. The contents of the gall-bladder, in the former disease, are usually abundant, fluid, and of a light colour; while in the latter they are scanty, dark-coloured, and consistent. The differences in the condition of the spleen, in the two diseases, are hardly less constant and striking. In half the subjects of yellow fever it is quite natural in all respects; in some it is slightly altered in volume or consistence; while, in remittent fever, it is found invariably very much enlarged or softened, or both." (pp. 385-86.)

Dr. Bartlett's opinion on the *vexata quæstio* of the contagion may be briefly stated. He considers, that in a pure atmosphere the disease is not transmissible from one individual to another; but that the unknown poison, wherever and in what way soever it may be generated, is capable of being carried in the holds of ships, and in close chests of clothing, from one place to another. Yellow fever, too, differs from remittent in the almost absolute immunity from a second attack which its occurrence confers on the individual. This immunity is supported by ample testimony.

The following is our author's concluding abstract of the points of difference between these two diseases,—yellow and remittent fever:

"They differ in their symptoms; their lesions; their causes; in the immunity from a second attack which is conferred by the occurrence of the former disease; in the preservative powers of *acclimatation;* and in duration. They are separate, individual diseases, each having its own characteristics; each, in most cases, easily distinguishable from the other; and each, in its several varieties, requiring its own mode of management." (p. 393.)

We have now given a view of the diagnostic portion of Dr. Bartlett's work. The distinction of the species and varieties of fever from each other being his principal object, he has not entered at any considerable length into the subject of treatment, except in the case of typhoid fever; and regarding this disease, the therapeutic methods adopted by some of the most eminent men in the United States and France, viz., Drs. Jackson and Nathan Smith in the former country, and MM. Chomel, Louis, Bouillaud, and De Larroque in the latter, are fully set forth. The plans of these distinguished parties are various enough. Those of the number, however, whose experience has been most extensive, Nathan Smith for example, and Chomel, think that mild cases cure themselves most surely without medication, all that is required being the administration of diluent drinks, a small quantity of farinaceous food, and the avoidance, as much as possible, of all causes of irritation. Bleeding is recommended, in the more inflammatory forms of the disease, by all the authorities quoted, excepting Dr. Jackson, whose chief reliance is on tartarized antimony, given first as an emetic, and afterwards in smaller but gradually increasing doses, according to the method of Odier, of Geneva. Bouillaud applies his plan of bleeding, *coup-sur-coup*, or (as Dr. Bartlett not inappropriately translates it) dash-upon-dash, to typhoid fever in common with all other acute diseases. On this practice our author remarks, that though Bouillaud has failed to establish the superiority of free and repeated bleeding in fever, yet he has shown that it is less dangerous than had been supposed,—in very plain English, he has killed fewer than might have been expected.

De Larroque trusts principally to purging, a proceeding which receives pretty strong approbation from Louis, in the second edition of his researches. Tonics and cordials are recommended under the circumstances which would naturally indicate their employment, by Chomel and Louis. The preparations of cinchona are the medicinal tonics employed, the former preferring the extract of bark, the latter sulphate of quinine. Mucilaginous enemata are recommended in the diarrhea, by Louis; and, should it in spite of these continue, he substitutes small enemata, containing a few drops of laudanum. Perforation of the intestine is treated, both by Louis and Chomel, according to the method of Drs. Graves and Stokes, with total abstinence and large doses of opium, though the doses recommended by Louis are smaller than those of the Dublin physicians.

The subject of treatment we consider a weighty one, especially in this country where the system of poly-pharmacy, we regret to say, prevails so much, perhaps in most diseases, certainly in fever. It seems to be overlooked that the tendency of fever in general, the typhoid variety not excepted, is to recovery, through a natural process, certainly a very exhausting one, but one which must be undergone; and it may be seriously questioned how far the perpetual administration of drugs—the *nimia medici diligentia*—does not injuriously disturb this process. Some of the most judicious and experienced of the writers quoted by Dr. Bartlett, Nathan Smith, for example, tells us that, in mild and simple cases, medicines, especially powerful ones, are injurious, and that the patient "gets along better" with diluent drinks, and a very small quantity of farinaceous food. Now our own experience tells us that not merely slight cases, but all cases "get along better" without medicines, excepting what may be required to empty the bowels at first, associated (perhaps), where the attack is a smart one, with one general bleeding; the diet mentioned by Nathan Smith, absolute repose in bed, a free supply of pure air, washing, and general cleanliness being rigidly observed. As a general rule, it is best to hold medicine, beyond the single purgative mentioned, in reserve, till the occasions for its employment occur.

What are these occasions? In the kind of fever now under consideration, they, in a great majority of instances, arise from the intestines, either in the form of diarrhea, hemorrhage, or perforation. Besides the mucilaginous drinks and enemata recommended by Louis for restraining the diarrhea, in this country at least, it will generally be found expedient to administer small doses of opium, combined with some astringent, such as chalk mixture. In hemorrhage from the intestines, which it has fallen to our lot to observe so frequently, and which we have not found so innocuous as it has appeared to Louis in France, we have resorted to strong astringents, sugar of lead, combined with opium, being the astringent which, in our hands, has proved the most effectual. In two cases in which the bleeding was so profuse as to threaten a fatal issue, all evacuations from the bowels were suspended for five days by means of the combination above mentioned. On the sixth day the bowels were moved by an enema of warm water; and this was continued daily till the intestines resumed their natural action. In these cases there was no recurrence of hemorrhage, and no subsequent untoward accident; convalescence after the lapse of some days took place without the employment of any other medicine. The diet which we have mentioned, and which we regard as the most important therapeutic agent in the disease, should be persevered in during the diarrhea and hemorrhage, excepting when this last takes place, as it sometimes does, to a sudden and great extent, producing great exhaustion, when food of a more nutritious and stimulating quality is required for a period, such as beef-tea and wine. With regard to the other intestinal accident, perforation, we know of no plan likely to save the patient but that of Graves and Stokes.

The other occasion for resorting to means not merely dietetic arises when, in the course of the disease, the head becomes affected, and there is considerable delirium. When this occurs in an excited and inflammatory form, with a hot skin and full pulse, leeches to the temples or nape, and cold epithems to the shaved scalp should be resorted to. There is another form of delirium observable generally in a more advanced stage of the disease, with extreme jactitation and restlessness, a small thready pulse of great rapidity, temperature of the skin even lower than in health, and with a contraction of the pupils, forming what Dr. Graves has called the pin-hole pupil. In its restlessness, tremor, and, frequently, its loquacity, this form of delirium exceedingly resembles delirium tremens. We have seen it successfully combated by opium in small doses frequently repeated. Dr. Graves advises belladonna in the same state, and our experience of this narcotic enables us to speak favorably of its powers; but we have derived equal advantage from opium. Tonics, especially the preparations of cinchona, recommended on the high authority of Chomel and Louis in the typhoid fever of France, have not appeared beneficial in fever of the same form in this country.

Great caution is required against deception by a pseudo-convalescence, which occurs more frequently in this fever than in any other. We should ever be suspicious of a convalescence which takes place before the third week. The cooling regimen has been adhered to, the diarrhea has been allayed by opiates and mild astringents; the skin becomes cool, especially in the morning, when the medical man probably makes his visit; there is sleep at night; the tongue is cleaner, and there is even some appetite. The friends of the patient are deceived, and so too, sometimes, is the medical attendant. From an idea of combating debility, some chicken or other solid food is given. In a day or two there is a recurrence of diarrhea, or, it may be, of hemorrhage; the abdomen, which had fallen, again becomes tympanitic, and the general feverish state of the system again lights up. An abatement of fever had been mistaken for its cessation; the solid food has irritated the intestinal affection, which was still existing amid the calm, and the whole process of treatment must again be repeated. Within our own experience, many lives have been lost in this way. We should recommend that convalescence should be well ascertained and established for some days before a change is made from farinaceous food to an animal diet. The smaller sorts of white sea-fish form an excellent stepping-stone from one to the other; but even *they* should not be taken till convalescence is fully established.

We cannot close this notice of Dr. Bartlett's work without formally expressing our opinion of its great value. Possessed of an ample acquaintance with the literature of his subject, he has not neglected to study it in that best of books, the book of nature. His brethren in the United States have much reason to thank him for his clear and compendious view of a class of diseases in which they must feel so deep an interest.

